# Toll- like receptor 2 polymorphism and IL-6 profile in relation to disease progression in chronic HBV infection: a case control study in Egyptian patients

**DOI:** 10.1007/s10238-022-00792-6

**Published:** 2022-02-04

**Authors:** Asmaa M. Elbrolosy, Naglaa S. Elabd, Gamalat A. ElGedawy, Mai Abozeid, Mervat Abdelkreem, Belal Montaser, Emad M. Eed, Moamena S. Elhamouly

**Affiliations:** 1 Medical Microbiology and Immunology Department, Faculty of Medicine, Menoufia University, Shebin El-Kom, 32511 Egypt; 2 Tropical Medicine Department, Faculty of Medicine, Menoufia University, Shebin El-Kom, 32511 Egypt; 3 Clinical Biochemistry and Molecular Diagnostics Department, National Liver Institute, Menoufia University, Shebin El-Kom, 32511 Egypt; 4 Hepatology and Gastroenterology Department, National Liver Institute, Menoufia University, Shebin El-Kom, 32511 Egypt; 5 Clinical Pathology Department, Faculty of Medicine, Menoufia University, Shebin El-Kom, 32511 Egypt

**Keywords:** HBV, TLR-2, SNPs, IL-6

## Abstract

Chronic hepatitis B (CHB) has a wide range of outcomes depending on host immune responses mainly Toll-like receptors (TLRs) signaling and released cytokines. Toll-like receptor 2 (TLR2) single nucleotide polymorphisms (SNPs) and interleukin 6 (IL-6) may influence the course of CHB. We aimed to elucidate the relation between TLR-2 polymorphism, IL-6 profile, and CHB progression. We analyzed TLR-2 polymorphism (SNP; rs3804099) in 185 CHB patients and 60 controls using TaqMan allelic discrimination assay. Serum IL-6 levels were assessed by ELISA. IL-6 levels were considerably higher in active CHB and cirrhotic patients compared with inactive carriers and controls (*P* < 0.001). IL-6 showed positive correlation with ALT and advanced fibrosis in active CHB patients (r = 0.31, *P* = 0.02). A significant positive correlation was noticed between IL-6 and HBV DNA PCR in all CHB groups. TT genotype of rs3804099/TLR-2 was significantly more prevalent in inactive carriers compared to active hepatitis patients (*P* = 0.04, OR = 0.39 and 95% CI: 0.16–0.95). Both heterozygous CT and mutant TT genotypes were significantly more frequent among inactive carriers compared to cirrhotic patients (*P* = 0.01, OR = 0.33, 95% CI: 0.13–0.81 and *P* = 0.009, OR = 0.32, 95% CI: 0.13–0.77). TT genotype was significantly related to lower IL-6 levels in active hepatitis and cirrhotic groups (*P* = 0.005 and *P* = 0.001, respectively) showing that TLR mutations would be associated with milder hepatitis activity and lower possibility for disease progression. There may be a positive association between TLR2 rs3804099 polymorphism and hepatitis B activity. IL-6 is a good indicator of CHB disease progression.

## Introduction

Hepatitis B virus (HBV) infection continues to be a serious health issue worldwide. Despite the availability of highly effective preventive vaccines and oral antivirals, the World Health Organization (WHO) recently estimated in 2019 that globally 296 million were infected with HBV and more than 820,000 deaths were attributed to HBV infection owing to the associated complications [1].

In the Mediterranean region, including Egypt, HBV genotype D is predominant and is often associated with HBeAg-negative variants that are linked with more severe liver disease [2, 3]. Egypt is burdened with approximately two to three million chronic HBV-infected persons. The highly prevalent HBeAg-negative variant in Egypt represents a late phase of HBV infection characterized by persistent viral replication, progression of liver disease and early cirrhosis development [4].

Hepatitis B virus is a hepatotropic virus that causes a persistent infection and triggers immune-mediated liver diseases of diverse duration severity [5]. The course of CHB is dynamic with fluctuations in HBV replication and liver inflammation. This variable course is governed by the complex interplay between viral replication and host immune control. The spectrum of HBV-induced liver disease ranges from an asymptomatic carrier state to chronic hepatitis, as well as cirrhosis and hepatocellular carcinoma (HCC) [6].

HBV differs in the way the virus interacts with the host immune system. It is well recognized that the outcome of HBV infection is influenced by both viral and host characteristics. The host defense against HBV involves both the innate as well as the adaptive systems. Innate immune receptors like toll-like receptors (TLRs), immune-regulatory cytokines and T-lymphocyte are the major components [7].

TLR-signaling pathways contribute to the pathogenesis and progression of several liver diseases including viral hepatitis, autoimmune liver disease, alcoholic and non-alcoholic liver disease, liver fibrosis, cirrhosis and hepatocellular carcinoma. TLR4 and TLR2 signaling have been suggested to be principal actors in inflammation, fibrosis and progression of chronic viral hepatitis. While TLR4 expression in hepatocytes is not upregulated by proinflammatory mediators, hepatocytes show increased responsiveness to TLR2 ligands under inflammatory conditions leading to up-regulation of TLR2 expression [8]. Toll-like receptors are pattern recognition receptors that recognize different viral components including envelope peptides, nucleocapsid and nucleic acids activating the immune cells and signaling pathways to enhance the release of proinflammatory cytokines and interferons [9]. Both circulating and intrahepatic innate immune system components can sense and respond to HBV infection. However, the robust response is accountable for hepatic necro-inflammation and liver damage [10].

The clinical relevance of TLR signaling has been highlighted by the identification of single nucleotide polymorphisms (SNPs) associated with the clinical course of chronic HBV infection [11]. The effects of TLRs gene variants in HBV-related liver disease are not well understood. TLR2 SNP (rs3804099) is a common genetic mutation site associated with viral liver disease in related publications [12–15]. It has been recently described to be associated with HBV disease activity in Chinese population [16].

IL-6 is considered one of the most important cytokines during HBV infection. It starts a cascade of signaling events promoting the transcription of multiple downstream genes associated with cellular signaling processes, including cytokines, receptors, adaptor proteins, and protein kinases. In addition to its roles modulating the host immune response, IL-6 has been implicated in the progression of several viral infections [17].

So, in this study, we aimed to elucidate the relation between TLR-2 promotor polymorphism (rs3804099), serum interleukin-6 (IL-6) profile and disease progression in CHB Egyptian patients.

## Patients and methods

This case–control study was carried out at departments of Hepatology and Gastroenterology, Clinical Biochemistry and Molecular Diagnostics, National Liver Institute (NLI) in collaboration with Tropical Medicine and Medical Microbiology and Immunology Departments, Faculty of Medicine, Menoufia University, Egypt between March 2020 and May 2021. During this study period, 185 Egyptian patients with chronic HBV infection (persistent positive HBsAg > 6 months with positive HBV-DNA by PCR) were recruited from outpatient clinics at NLI and Tropical Medicine department, Menoufia University. Besides, 60 age and sex-matched healthy volunteers served as a control group. Exclusion criteria were acute HBV infection, hepatitis C virus (HCV) or human immunodeficiency virus (HIV) co-infection and other causes of liver diseases including non-viral etiologies (e.g., metabolic liver diseases, non-alcoholic fatty liver disease (NAFLD) and autoimmune liver diseases). Patients with history of acute or chronic inflammatory disease in the preceding 3 months, obesity, diabetes mellitus, impaired glucose tolerance (prediabetes), malignancies or autoimmune diseases were precluded from the study.

Concerning each patient, history was assessed together with detailed clinical evaluation. All participants were tested for the presence of HBsAg as well as HCV and HIV antibodies using ELISA. Additionally, HBV viral load was quantitatively estimated by real-time PCR. Other laboratory investigations including, liver function tests [international normalized ratio (INR), ALT, aspartate transaminase (AST), direct bilirubin (DBIL), total bilirubin (TBIL), serum albumin and serum α-fetoprotein (AFP), complete blood count, were assessed.

Imaging evaluation was performed for all participants by abdominal ultrasound and the stage of liver fibrosis was assessed by transient elastography (Fibroscan) for all patients. Child-Turcotte-Pugh score was calculated for cirrhotic patients to assess the liver disease severity [18].

The study participants were categorized into four groups as per European Association for the Study of the Liver (EASL) [19].

### Group I

Included 65 patients with inactive chronic HBV infection [inactive carrier state, defined by normal alanine aminotransferase (ALT), low viral replication (HBV DNA < 2000 IU/mL) and no liver fibrosis] without liver cirrhosis or evidence of liver tumors. All selected patients did not previously receive antiviral treatment.

### Group II

Included 62 patients with active chronic HBV infection [HBeAg-negative chronic hepatitis, defined by persistently elevated ALT and high viral replication (HBV DNA > 2000 IU/mL) and significant liver fibrosis] without liver cirrhosis or evidence of liver tumors.

### Group III

Included 58 patients with liver cirrhosis secondary to chronic HBV infection (diagnosis of cirrhosis was done by history, clinical evaluation, laboratory investigations and ultrasound and fibroscan findings).

### Group IV

Included 60 sex and age-matched healthy volunteers with no history of previous liver disease, negative HBsAg and HCVAb and normal liver function tests as a control group.

### Measurement of IL-6 serum level

Human IL-6 quantikine ELISA kits (double antibody sandwich enzyme-linked immunosorbent assay, SunRed, China, Cat.No.201–12-0091) were used to measure the serum level of IL-6. Firstly 50 μl of assay diluent were pipetted to each well, then 200 μl of standard, control or serum sample (100 μl) were pipetted to each well. Plates were covered with plate sealer and incubated at room temperature for 2 h. Each well was aspirated and washed, repeating the process 3 times for a total of 4 washes. About 200 μL of conjugate were added to each well then covered with a new plate sealer, and incubated at room temperature for 2 h on the shaker then wells were aspirated and washed for 4 times. About 200 μL substrate solution was added to each well, incubated at room temperature for 30 min on the bench top, and kept from the exposure of light. About 50 μL of stop solution were added to each well. Results were read at 450 nm within 30 min. A standard curve was drawn and the IL-6 concentrations were determined for each sample by using the manufacturer’s software.

### Characterization of Toll-like receptor-2 promotor polymorphism

SNP genotyping for rs3804099 was performed by TaqMan allelic discrimination assay which is an endpoint assay (data are collected at the end of Polymerase Chain Reaction process) that detects variants of a single nucleic acid sequence. The existence of the two primer/probe pairs in each reaction governs genotyping of the two possible variants at the SNP site in a target template sequence with allele-specific fluorogenic oligonucleotide probes. The allelic discrimination assay categorizes unknown samples as follows: A. Homozygotes: samples with only allele 1 or allele 2. B. Heterozygotes: samples with both allele 1 and allele 2.

Human genomic DNA was extracted from venous EDTA- anticoagulated whole blood by using a spin column method according to the manufactures instructions (Thermo Scientific, Lithuania, Gene jet whole blood genomic DNA purification Mini kit). Reaction qPCR Master Mix (2 ×) for amplification (total volume 20 μl) constituted of: 0.5 μl of genotyping assay (primer/probe mix), 10 μl of genotyping qPCR Master Mix (Thermo Fisher Scientific, MA, USA), 3.5 μl of DNAse-free water and 6 μl (0.1 μg/μl) of genomic DNA template was added. For the negative control reaction, 6 μl of DNAse-free water was added. The PCR and genotyping were performed on real time fast 7500 (Thermo Fisher Scientific Inc., Life Technologies TM, CA, USA). The cycling parameters were set as follows: Pre-PCR (holding stage): 60 °C for 1 min, initial denaturation step: 95 °C for 10 min, cycling stage: denaturation step: 95 °C for 1 min, annealing and extension: 60 °C for 1 min and post-PCR (holding stage): 60 °C for1 minute.

### Statistical data analysis

Data of this study were coded, entered and analyzed by using SPSS (Statistical Package for Social Science) version 20.0 on IBM compatible computer (SPSS Inc., Chicago, IL, USA). Qualitative data were described as frequency and percentage and analyzed using chi square test, quantitative data were described as mean, standard deviation, median and range and were analyzed between the four studied groups using ANOVA test when the data was normally distributed and Kruskal Wallis test was used to compare multiple groups with normally distributed data and post hoc test was used to compare the groups in pairs, Spearman correlation was performed to correlate IL-6 and other quantitative parameters, The crude OR measured the risk of exposure to mutant genotypes. Regression analysis is a statistical process for estimating independent risk for progression of liver disease, P value was considered significant when less than 0.05.

## Results

This case–control study involved 245 participants. They were categorized into four groups; Group I: Included 65 patients with inactive chronic HBV infection of which 29 (44.6%) were males and 36 (55.4%) were females with a mean age of 52.5 ± 11.8 years. Group II: Comprised 62 patients [40 (64.5%) males and 22 (35.5%) females] with active CHB with a mean age of 55.3 ± 9.4 years. Group III: Included 58 patients liver cirrhosis secondary to chronic HBV infection of which 35 (60.3%) were males and 23 (39.7%) were females with a mean age of 54.5 ± 11.7 years. Group IV: Involved 60 age and sex-coincided healthy controls. No difference was observed between the four studied groups regarding age and sex. ALT, AST, INR, serum albumin, total bilirubin, and AFP were significantly different amongst the four studied groups (*P* < 0.001). Higher ALT, AST levels were observed in groups II and III and lower albumin levels were found in cirrhotic patients as displayed in Table 1. Platelet count and hemoglobin concentration showed considerably lower levels in group III cirrhotic patients. However, no difference was noted regarding total leukocyte count between the studied groups (Table 1).

**Table 1:** Comparison between the studied groups regarding demographic data, clinical data, laboratory parameters and serum IL-6 level

Variables	Group I (n = 65)	Group II (n = 62)	Group III (n = 58)	Group IV (n = 60)	*P*-value (*P*)
*Age*					
Mean ± SD Median (min.–max.)	52.5 ± 11.8 54 (29–70)	55.3 ± 9.4 54 (32–71)	54.5 ± 11.7 56 (37–73)	55.6 ± 10.4 55 (37–73)	F = 1.03 P = 0.38
*Gender*					
Male Female	29 (44.6%) 36 (55.4%)	40 (64.5%) 22 (35.5%)	35 (60.3%) 23 (39.7%)	35 (58.3%) 25 (41.7%)	X^2^ = 5.79 P = 0.12
*ALT (IU/L)*					
Mean ± SD Median (min.–max.)	26.3 ± 8.0 29 (11–36)	111.2 ± 47.3 88 (71–241)	94.0 ± 56.5 71.5 (49–215)	15.9 ± 4.5 15 (7–24)	K = 199 P < 0.001
Post hoc		< 0.001^1^	< 0.001^1^ 0.37^2^	< 0.001^1^ < 0.001^2^ < 0.001^3^	
*AST (IU/L)*					
Mean ± SD Median (min.–max.)	28.2 ± 7.2 28 (13–37)	93.1 ± 42.5 79 (56–210)	108.7 ± 70.1 81.5 (57–274)	21.5 ± 5.3 22.5 (13–35)	K = 190 P < 0.001
Post hoc		< 0.001^1^	< 0.001^1^ 0.62^2^	< 0.001^1^ < 0.001^2^ < 0.001^3^	
*Total bilirubin (mg/dl)*					
Mean ± SD Median (min.–max.)	0.82 ± 0.19 0.9 (0.4–1.0)	0.91 ± 0.32 0.9 (0.4–1.6)	1.3 ± 0.88 1.1 (0.4–3.4)	0.9 ± 0.16 0.9 (0.7–1.2)	K = 100.48 P < 0.001
Post hoc		0.23^1^	< 0.001^1^ 0.005^2^	0.65^1^ 0.80^2^ 0.001^3^	
*Serum albumin (gm/dl)*					
Mean ± SD Median (min.–max.)	4.63 ± 0.29 4.6 (4.1–5)	4.59 ± 0.31 4.7 (3.8–4.9)	3.39 ± 0.69 3.5 (2.3–4.5)	4.69 ± 0.32 4.6 (4–5.3)	F = 124 P < 0.001
Post hoc		0.59^1^	< 0.001^1^ < 0.001^2^	0.41^1^ 0.18^2^ < 0.001^3^	
*HBV-DNA PCR (IU/ml)*					
Mean ± SD Median (min.–max.)	637.6 ± 543.23 562 (10–1626)	12,779 ± 9045.2 9630 (3400–35,700)	53,146.9 ± 99,099.7 11,768.5 (3800–330,800)	–	K = 127.1 P < 0.001
Post hoc		< 0.001^1^	< 0.001^1^		
			0.009^2^		
*INR*					
Mean ± SD Median (min.–max.)	1.02 ± 0.06 1.0 (1.0–1.2)	1.03 ± 0.05 1 (1–1.1)	1.26 ± 0.21 1.2 (1–1.7)	1.01 ± 0.08 1.0 (0.9–1.1)	F = 60.91 P < 0.001
Post hoc		0.88^1^	< 0.001^1^ < 0.001^2^	0.55^1^ 0.46^2^ < 0.001^3^	
*Platelets (X 10*^*3*^*)*					
Mean ± SD Median (min.–max.)	242.86 ± 43.31 233(170–320)	231.66 ± 40.1 225 (187–301)	173.67 ± 47.77 180 (89–254)	242.9 ± 58.64 245 (165–400)	F = 28.41 P < 0.001
Post hoc		0.19^1^	< 0.001^1^ < 0.001^2^	0.99^1^ 0.20^2^ < 0.001^3^	
*Hb (gm/dl)*					
Mean ± SD Median (min.–max.)	14.0 ± 1.2 14 (12.1–16.1)	13.6 ± 1.3 13.3 (11.9–16)	12.7 ± 1.4 12.4 (11.3–16.6)	13.9 ± 0.9 14 (12.5–15)	F = 15 P < 0.001
Post hoc		0.04^1^	< 0.001^1^ < 0.001^2^	0.42^1^ 0.23^2^ < 0.001^3^	
*WBCs (X 10*^*3*^*)*					
Mean ± SD Median (min.–max.)	6.5 ± 1.5 6.6 (4.6–8.9)	6.6 ± 1.3 6.3 (4.9–9.3)	6.0 ± 1.3 6.1 (3.7–8.4)	6.3 ± 1.8 5.85(4.2–11)	F = 2.18 P = 0.09
Post hoc		0.73^1^	0.04^1^ 0.02^2^	0.45^1^ 0.28^2^ 0.21^3^	
*AFP (IU/ml)*					
Mean ± SD Median (min.–max.)	2.09 ± 1.2 1.68(0.7–4.7)	4.9 ± 3.0 4 (1.8–11.8)	10.5 ± 4.9 9.65 (2.3–20.5)	2.65 ± 1.1 3 (1–4)	F = 101.6 P < 0.001
*IL-6 (pg/ml)*					
Mean ± SD Median (min.–max.)	41.3 ± 10.7 43 (15–56)	74.9 ± 23.8 77.5 (19–115)	133.9 ± 20.1 138(92–174)	15.0 ± 3.3 14.5 (10–21)	F = 575.2 P < 0.001
Post hoc		< 0.001^1^	< 0.001^1^ < 0.001^2^	< 0.001^1^ < 0.001^2^ < 0.001^3^	
*Fibrosis stage (Fibroscan)*					
F0 F1 F2 F3 F4	45 (69.2%) 20 (30.8%) 0 (0%) 0 (0%) 0 (0%)	0 (0%) 7 (11.3%) 36 (58.1%) 19 (30.6%) 0 (0%)	0 (0%) 0 (0%) 0 (0%) 0 (0%) 58 (100%)		X^2^ = 339.7 P < 0.001

*ALT* alanine aminotransferase; *AST* aspartate aminotransferase, *INR* international normalized ratio, *HB* hemoglobin concentration, *WBCs* white blood cells, *AFP* serum α-fetoprotein. *f*  ANOVA test, *K* Kruskal Wallis test, *X*^*2*^ Chi square test

^1^Comparison with group 1

^2^Comparing with group 2

^3^Comparison with group 3

**Fig. 1:**
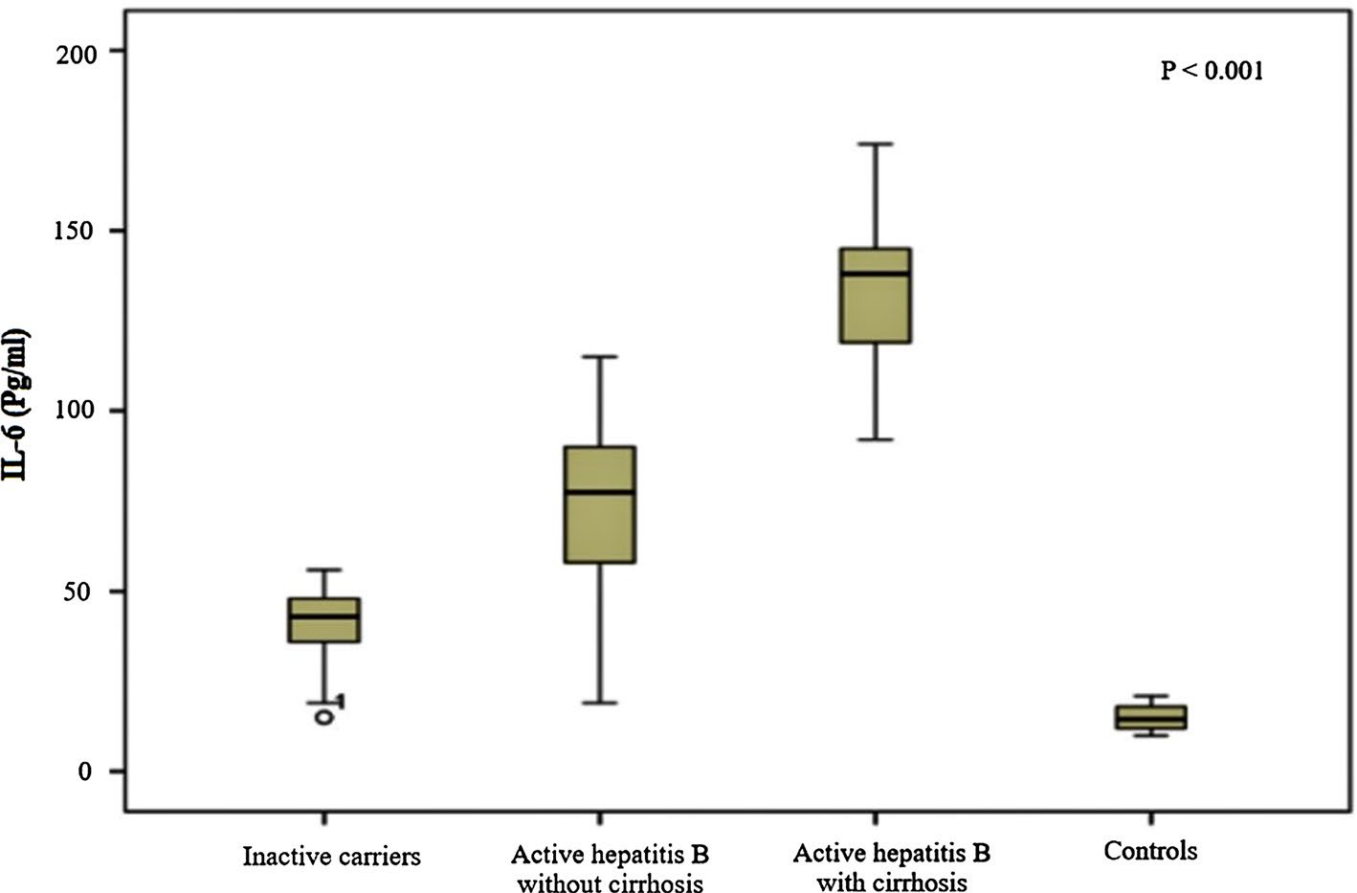
IL-6 (pg/ml) among the studied groups

**Fig. 2:**
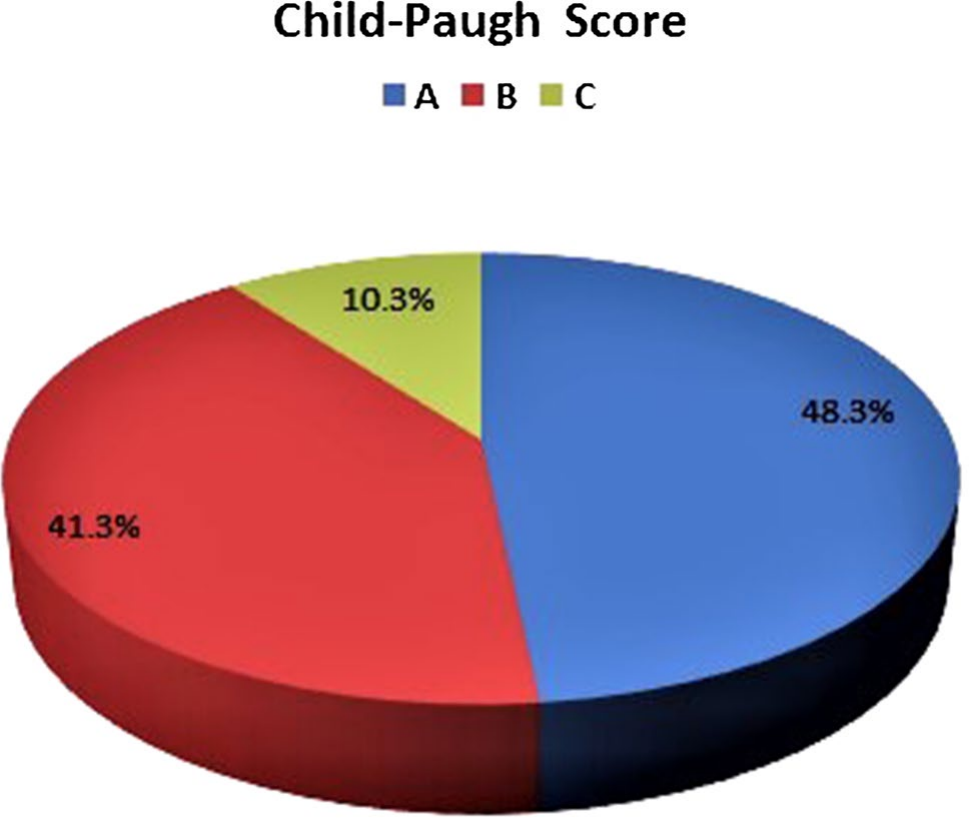
Child- Paugh score among cirrhotic liver group

IL-6 showed considerably higher values in groups II and III compared with those detected in groups I and IV (*P* < 0.001) with higher levels in cirrhotic patients compared with chronic HBV patients (*P* < 0.001). Additionally, a significant difference was found between patients with active CHB in relation to inactive carriers and controls (*P* < 0.001) as shown in Table 1 and Fig. 1. Fibrosis stages F1, F2 and F3 accounted for 11.3%, 58.1%, and 30.6%, respectively for group II patients as demonstrated by Fibroscan. Child-Paugh Score of the cirrhotic group was presented in Fig. 2.

**Fig. 3:**
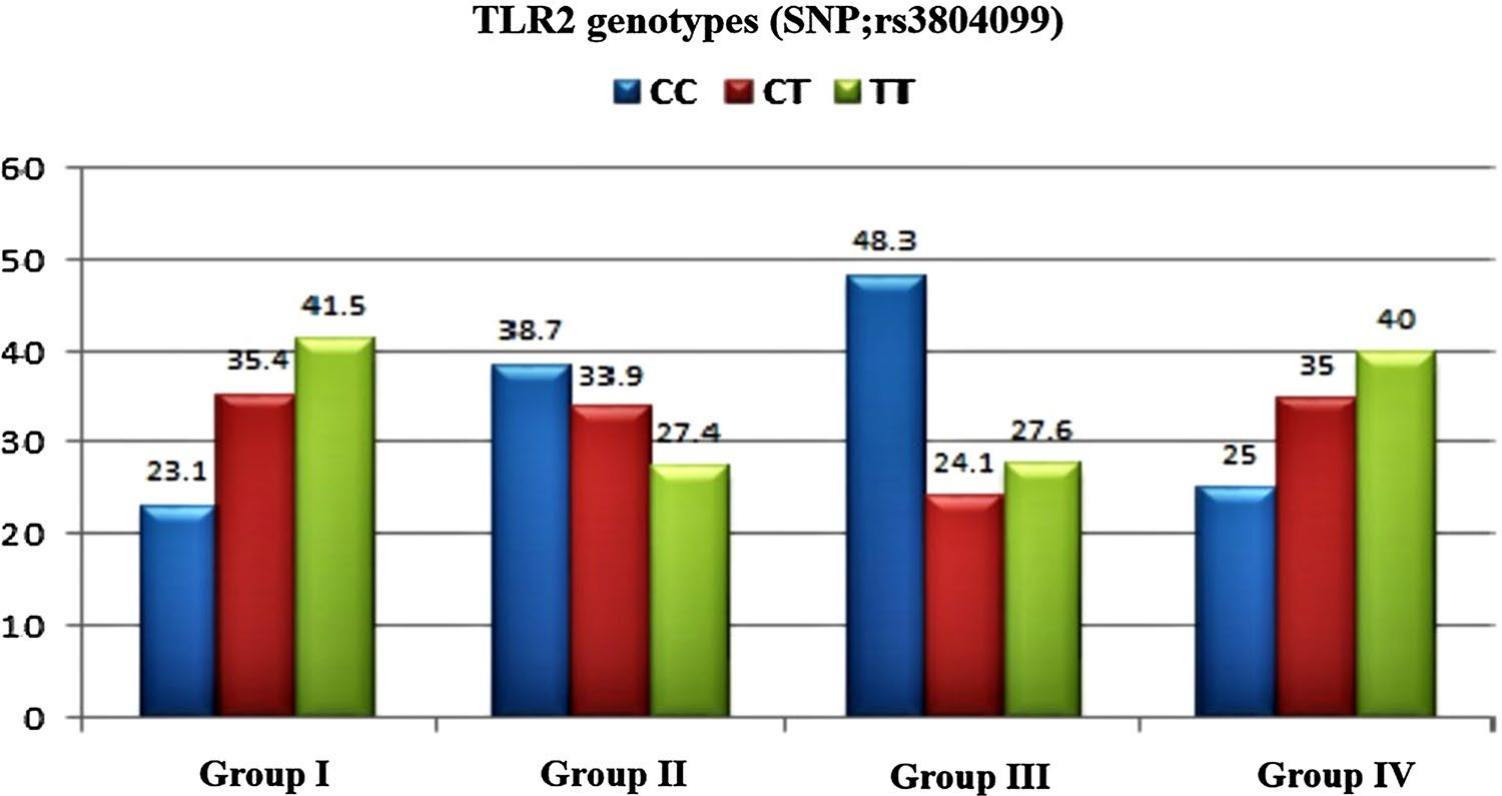
TLR2 genotypes (SNP; rs3804099) genotypes among the studied groups

Table 2 displays genotypes frequencies and allelic distribution for rs3804099 of TLR-2 polymorphism among the studied groups. Importantly, we observed that TT genotype was more prevalent in asymptomatic carrier group (41.5%) compared to 27.4% for patients with active CHB with a significant statistical difference (*P* = 0.04, OR = 0.39 and 95% CI: 0.16–0.95). Similarly, the T allele showed the same distribution (59.2.5 vs. 47.6%, respectively, for group I and group II; *P* = 0.02, OR = 0.55 and 95% CI: 0.33–0.90). Additionally, both heterozygous CT and mutant TT genotypes were significantly more frequent among inactive carrier group as compared to cirrhotic group (*P* = 0.01, OR = 0.33 & 95% CI: 0.13–0.81 and *P* = 0.009, OR = 0.32 and 95% CI: 0.13–0.77) giving more evidence that TLR mutations reduces the possibility of disease progression in chronic HBV infected patients. However, the genotype and allelic frequencies between active hepatitis and cirrhotic groups was not significantly different (P > 0.05) as shown in Fig. 3.

**Table 2:** Genotypes frequencies and allelic distribution for TLR-2 polymorphism among the studied groups

TLR2 genotypes (SNP;rs3804099)	Group I (n = 65)	Group II (n = 62)	*X*^*2*^ Test 1 *P* value	Odds ratio 1 95% CI	Group III (n = 58)	*X*^*2*^ Test 2 *P* value	Odds ratio 2 95% CI	*X*^*2*^ Test 3 *P* value	Odds ratio 3 95% CI
CC	15 (23.1)	24 (38.7)		**Ref (1)**	28 (48.3)		**Ref (1)**		**Ref (1)**
CT	23 (35.4)	21 (33.9)	1.59 0.21	0.57 0.24–1.37	14 (24.1)	5.93 0.01	0.33 0.13–0.81	1.61 0.21	0.57 0.24–1.36
TT	27 (41.5)	17 (27.4)	4.34 **0.04**	0.39 **0.16–0.95**	16 (27.6)	6.7 **0.009**	0.32 **0.13–0.77**	0.23 0.63	0.81 0.34–1.93
**Alleles**	**N = 130**	**N = 124**			**N = 116**				
C	53 (40.8)	69 (55.6)		Ref (1)	70 (60.3)		**Ref (1)**		**Ref (1)**
T	77 (59.2)	55 (47.6)	5.63 **0.02**	0.55 **0.33–0.90**	46 (39.7)	9.4 **0.002**	0.45 **0.27–0.75**	0.54 0.46	0.82 0.49–1.38

*CC* wild type, *CT* heterozygous, *TT* homozygous, *X*^*2*^ Chi square test

^1^Comparing Group 1 and group II with group I was a reference group

^2^Comparing Group 1 and group III with group I was a reference group

^3^Comparing Group II and group III with group II was a reference group

**Fig. 4:**
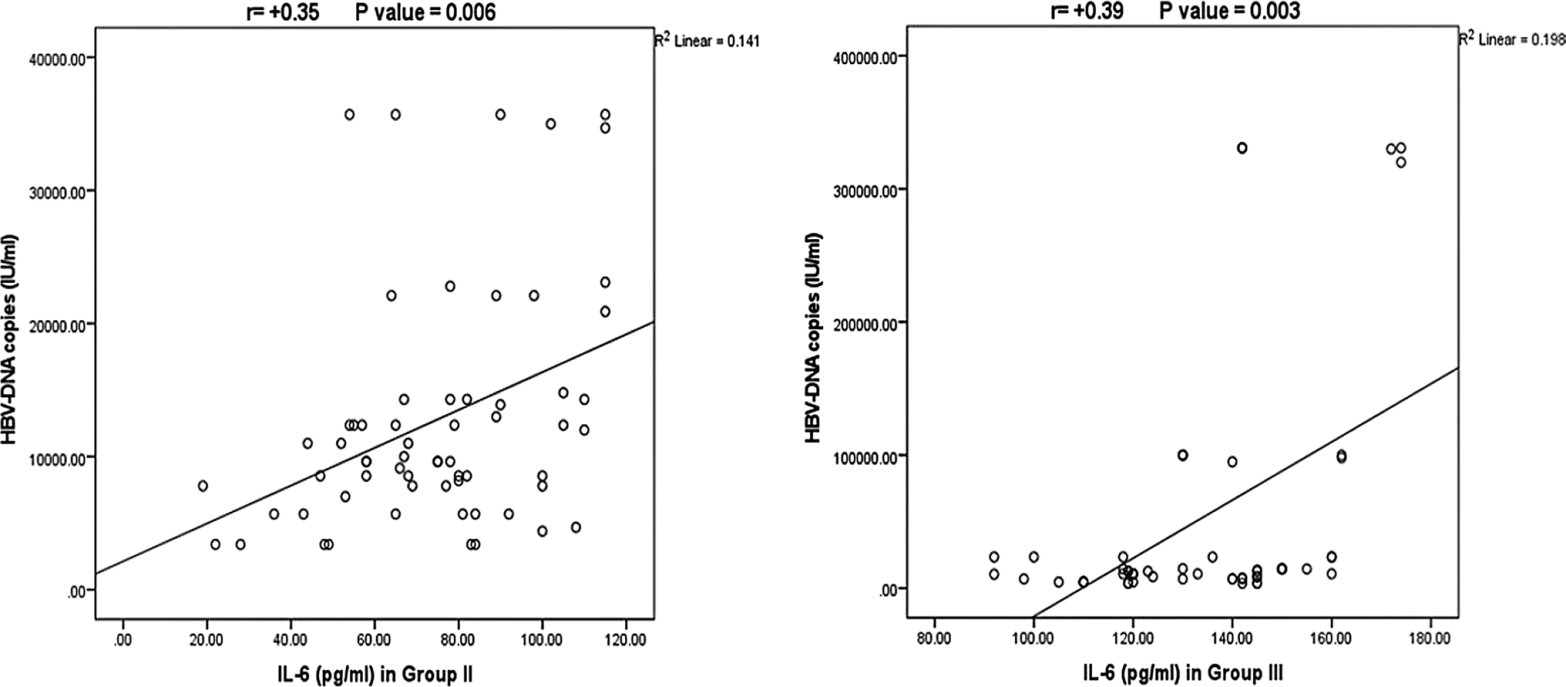
Correlation between IL-6 levels and HBV-DNA copies in group II and III

Regarding the correlation between IL-6 levels and disease activity parameters among CHB groups, as presented in Table 3, we noticed a significant positive correlation between IL-6 levels and ALT, AST and advanced fibrosis stage [(r = 0.31, *P* = 0.02), (r = 0.28, *P* = 0.03) and (r = 0.31, *P* = 0.02), respectively] in patients with active CHB. Moreover, higher IL-6 levels were positively correlated with higher HBV-DNA PCR (IU/ml) in groups I, II and III [(r = 0.26, *P* = 0.04), (r = 0.35, *P* = 0.006) and (r = 0.39, *P* = 0.003), respectively] (Fig. 4). For group III cirrhotic patients, higher IL-6 values significantly correlated with Child Pugh score (r = 0.39, *P* = 0.002) and with lower platelet count (r = −0.26, *P* = 0.045).

**Table 3:** Correlation between IL-6 profile and disease activity parameters among CHB groups

Variables	Group I (n = 65)		Group II (n = 62)		Group III (n = 58)	
	r	*P*-value	r	*P*-value	r	*P*-value
Age	−0.008	0.95	0.18	0.16	0.23	0.09
ALT (IU/L)	−0.22	0.07	0.31	0.02	−0.05	0.72
AST (IU/L)	−0.18	0.16	0.28	0.03	−0.04	0.74
Total bilirubin (mg/dl)	0.14	0.28	−0.08	0.54	0.002	0.99
Albumin (gm/dl)	0.17	0.17	−0.11	0.41	0.04	0.79
HBV-DNA copies (IU/ml)	0.26	0.04	0.35	0.006	0.39	0.003
INR	0.04	0.74	−0.07	0.59	−0.08	0.57
Platelet count (X 103)	−0.31	0.01	−0.15	0.26	−0.26	0.045
Hb (gm/dl)	0.22	0.08	0.03	0.81	0.38	0.004
WBCs (X 103)	0.05	0.71	−0.13	0.30	0.09	0.53
Fibrosis stage	0.28	0.03	0.31	0.02	–	–
Child-Paugh Score	–		–	–	0.39	0.002

*ALT* alanine aminotransferase, *AST* aspartate aminotransferase, *INR* international normalized ratio, *HB* hemoglobin concentration, *WBCs* white blood cells

**Fig. 5:**
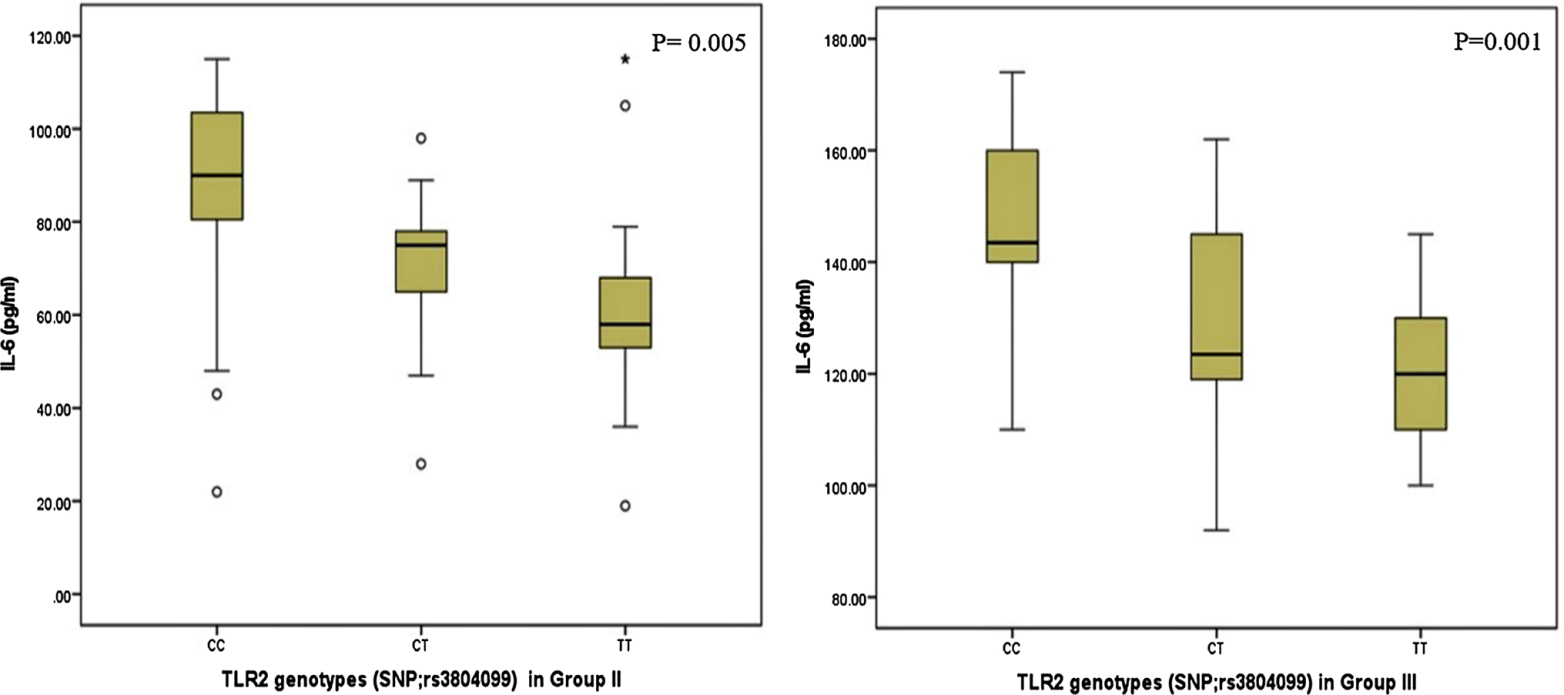
Relation between TLR2 genotypes and IL-6 levels in group II and III

According to Table 4, there was a significant relation between genetic variants of TLR-2 and disease activity parameters in CHB patients. For both active hepatitis and cirrhotic groups, patients carrying the wild CC genetic variant had higher levels of IL-6 with a significant statistical difference compared to those carrying the heterozygous CT and mutant TT genotypes [P = 0.005 and *P* = 0.001, respectively] (Fig. 5). Moreover, genetic variants of TLR-2 related significantly to Child-Paugh Score, as all patients with C score carried the wild CC variant with a significant statistical difference (*P* = 0.02). As a marker of disease activity and progression, we observed that the mean values of ALT enzyme were significantly lowered in patients with active hepatitis who carried mutant TT variant as compared to wild type (*P* = 0.04).

**Table 4:** Correlation between rs3804099 genotypes of TLR2 and disease activity parameters and IL-6 profile among CHB groups

	TLR2 genotypes (SNP; rs3804099) Group II			Test	*P* value	TLR2 genotypes (SNP; rs3804099) Group III			Test	*P* value
	CC N = 24	CT N = 21	TT N = 17	K		CC N = 28	CT N = 14	TT N = 16	K	
*AST* Mean ± SD Median Range	79.0 ± 33.48 68 56–210	109.09 ± 52.16 85 67–210	93.35 ± 34.13 90 56–205	8.17	0.01	104.39 ± 66.44 64 57–274	81.14 ± 11.78 84 64 – 92	140.44 ± 93.67 88.5 60 – 274	4.57	0.10
*ALT* Mean ± SD Median Range	105.0 ± 46.6 86.5 71–241	129.57 ± 58.35 122 78–241	97.23 ± 20.87 88 71–125	4.97	0.10	92.39 ± 58.66 61 49–215	73.93 ± 13.67 75 55 – 88	114.37 ± 70.42 74 53 – 215	4.28	0.12
*Viral load* Mean ± SD Median Range	12,339.9 ± 11,173.4 8000 3400—35,700	13,103.7 ± 7710.1 9630 3400–35,700	12,997.8 ± 7347.2 11,000 5688–35,700	2.70	0.26	85,987.2 ± 131,198.1 14,500 3800–330,800	16,968.6 ± 23,604.1 12,720 3800—98,000	26,770.1 ± 36,858.7 10,817 3800—100,000	2.54	0.28
*Fibrosis* stage Stage 1 Stage 2 Stage 3	1 (4.2) 15 (62.5) 8 (33.3)	3 (14.3) 12 (57.1) 6 (28.6)	3 (17.6) 9 (52.9) 5 (29.4)	X2 2.11	0.72					
*Child Paugh* A B C						10 (35.7) 12 (42.9) 6 (21.4)	6 (42.9) 8 (57.1) 0 (0.0)	12 (75.0) 4 (25.0) 0 (0.0)	X2 11.7	0.02
*IL-6* Mean ± SD Median Range	85.29 ± 25.06 90 22–115	71.28 ± 15.08 75 28–98	64.65 ± 26.09 58 19–115	10.43	0.005	142.85 ± 20.27 142 92–174	130.71 ± 18.91 123.5 92–162	121.19 ± 12.1 120 100–145	13.4	0.001

*k* Kruskal wallis test, *X2 * Chi square test

Multivariate regression analysis revealed in model 1 that IL-6 and TLR2 genotypes (SNP; rs3804099) were independent risk factors for progression of liver disease from inactive carrier state to active hepatitis state (*P* = 0.03, OR = 2.5 and 95% CI: 1.4–6.68) and (*P* = 0.008, OR = 0.65 and 95% CI:0.08–1.13, respectively). Meanwhile, viral load, IL-6 and TLR2 genotypes (SNP; rs3804099) were independent risk factors for progression of liver disease from active hepatitis state to liver cirrhosis with (*P* = 0.005, OR = 1.78, 95% CI: 1.12–4.54), (*P* < 0.001, OR = 2.68, 95% CI: 1.4–9.18) and (*P* < 0.001, OR = 0.5, 95% CI: 0.17–2.08), respectively, (Table 5).

**Table 5:** Multivariate regression analysis for independent risk factors affecting progression of liver disease

Model 1	SE	Wald X2	*P* value	Odds ratio	95% CI
Viral load	1.88	1.23	0.23	1.13	0.10–7.44
IL-6	1.01	2.18	0.03	2.5	1.4–6.68
TLR2	1.44	2.71	0.008	0.65	0.08–1.13

Model 2	SE	Wald X2	*P* value	Odds ratio	95% CI
Viral load	1.17	2.33	0.005	1.78	1.12–4.54
IL-6	2.41	2.78	< 0.001	2.68	1.4–9.18
TLR2	3.98	3.11	< 0.001	0.5	0.17–2.08

Model 1: independent risk factors for progression from inactive carrier to active CHB

Model 2: independent risk factors for progression from active CHB to liver cirrhosis

*SE * standard error, *CI * confidence interval

## Discussion

Hepatitis B virus infection can cause chronic hepatitis that has long term complications with great variability in CHB disease progression rates [20]. Both HBV-related liver damage and viral control are thought to be immune-mediated [21]. In HBV-infected patients, abnormal numbers and dysfunctions of T cells have been found to be closely related to development, chronicity and progression of HBV [22]. There is growing evidence that cytokine-mediated immune responses play a vital role in determining HBV clinical outcomes [23].

Toll-like receptors are transmembrane receptors that govern the immune response against microbes and cytokine gene expression. TLR-2 activation may be involved in the progression of numerous diseases including viral hepatitis B [24]. Engagement of TLRs with viral epitopes will activate a signaling cascade to mediate the release of proinflammatory cytokines.IL-6 is considered as the main mediator of the inflammation and acute phase responses of the liver as well, it could mediate chronic disease progression via saving T cells from apoptosis during the inflammatory process [17].

HBV contains TLR ligands and induces cytokine production via the TLR2 signalling pathway [25]. Little is recognized about the effect of TLR2 polymorphism in chronic HBV progression. TLR2 SNP (rs3804099) is a commonly recognized genetic mutation site in several studies mainly on Asian populations infected with HBV [12–14, 16]. It has been recently described to be associated with the outcome of HCV infection, response to therapy and development of HCC in Egyptian patients [15]. We conducted this study to verify the association of SNP (rs3804099) of TLR2 and IL-6 with disease activity and progression of chronic HBV infection in Egyptian population.

In the current study, considerably significant higher values were detected regarding serum level of IL-6 in CHB patients compared to healthy controls. Importantly, the current results also proved that serum IL-6 profile correlated well with the degree of liver affection among the studied groups. Patients with HBV-related liver cirrhosis exhibited more elevated IL-6 levels than those with active chronic HBV. In addition, serum samples from chronic active hepatitis B group yielded higher IL-6 values than those with inactive carrier state. IL-6 is reported to be highly expressed in the early stage of acute and chronic liver injury [26]. It initiates warning signals to the entire body, and many experiments have reported that the serum levels IL-6 are elevated in CHB patients [27]. Being a mediator of inflammation, IL-6 may be implicated in the progression of HBV-associated liver cirrhosis [28]. Previously, it was documented that IL-6 is capable of suppressing replication of HBV together with inhibiting the accumulation of HBV-covalently closed circular DNA (ccc DNA) in human hepatoma cells [29]. By contrast, another study has notified increased serum IL-6 levels in chronic HBV infected patients who developed HCC [28]. It has been reported that reducing viral load and thereby reducing the IL-6 by anti-HBV treatment have a certain role in promoting functional recovery of liver cells further indicating that IL-6 measures are closely linked with the extent of liver cell necrosis [30]. In the same context, Alison et al. documented that upregulated IL-6 expressions further activate a corresponding inflammation-related signaling pathway that in turn drives hepatitis B progression to cirrhosis or HCC [31]. Additionally, in former research, Xia et al. verified that IL-6 levels increase with hepatitis B Virus X protein (HBx) expressions in the hepatocytes, and that the underlying mechanism for the HBx-induced production of IL-6 occur in a MyD88- (myeloid differentiation factor 88) dependent manner [32]. Furthermore, it has been concluded that, in an HBV-infected liver microenvironment the parenchymal liver cells are additional sources of high IL-6 levels. Therefore, HBx is considered to be an IL-6 upregulator that promote development of cirrhosis via an IL-6-mediated increase in expression of microRNA-21 [33].

The present study exhibited remarkable associations between IL-6 levels with evidence of HBV-related disease severity including ALT, AST, advanced fibrosis stage, increased Child Pugh score, and lower platelet count. It was formerly stated for CHB patients, IL-6 levels were significantly higher in patients with severe compared those with mild to moderate inflammation as well as the healthy control groups; besides, IL-6 showed a positive correlation with ALT, AST and TBIL as indicators of liver function, so, they documented that the level of serum IL-6 was closely related to the degree of necrosis of liver cells [11]. Similar results were also reported regarding the correlations of IL-6 levels with biochemical markers of liver disease [34, 35] reflecting its role in evaluating the degree of activity of the inflammatory process in the liver. In their study about serum levels of IL-6 in HBV-induced Child–Pugh B cirrhosis Cai et al. recommended monitoring of changes in levels of IL-6 for evaluating the prognosis of CHB patients with cirrhosis [36]. We displayed remarkable positive associations between IL-6 levels and HBV-DNA PCR. These results agree with previous literature, where IL-6 positively correlated with HBV viral load. They also reported, owing to the fact that increased HBV vital load may further lead to increased cellular immune dysfunction in HBV patients, there by producing and releasing IL-6 to promote inflammatory responses [34].

There has been substantial research undertaken on the effect of TLRs-related immune defense against various pathogens, such as HBV [37]. Nevertheless, current findings revealed that genetic mutation and polymorphisms in the promoter region of TLR2 correlated with milder CHB progression. The concept of TLR2 activation as a protective mechanism has been recently challenged. Consistent with the literature, better liver assessment parameters were observed among Egyptian population with TLR2 mutant variants. We observed that chronic HBV infected patients carrying heterozygous CT or mutant TT genotypes of rs3804099 of TLR2 had milder disease activity than those carrying the wild type CC variant. Moreover, heterozygous CT and mutant TT genotypes were associated with lower levels of hepatic markers of HBV activity particularly ALT levels. On the other hand, those with CC wild genotype expressed higher levels of liver enzymes and showed more aggressive disease activity. Ultimately, genetic variants of TLR-2 related significantly to Child-Paugh Score among cirrhotic patients. Higher percentage of patients with C score carried the wild CC variant providing more evidence for the association between TLR genetic mutation and progression of liver affection. Evermore, multivariate regression analysis of the current findings revealed that IL-6 and TLR2 genotypes (SNP; rs3804099) were independent risk factors for progression of liver disease from inactive carrier state to active hepatitis state. Meanwhile, viral load, IL-6 and TLR2 genotypes were independent risk factors for progression of liver disease from active hepatitis state to cirrhotic liver disease. Lin et al. also found that TLR2 mutations were considerably linked to milder hepatitis activity among chronic HBV infected patients and concluded that TLR pathways activation may additionally intensify the hepatocyte inflammation and leads to progression of disease that was partially in accordance with our findings. Moreover, close associations were found between rs3804099 polymorphisms of TLR2 and CHB outcomes pointing out that individuals carrying the heterozygous genotypes of this SNP were at minimized risk of disease progression than those carrying the wild-type homozygous genotype [12, 38].

We assumed that polymorphisms could affect the signaling of TLR2 to hepatocytes, particularly concerning inflammation status. Mutations are probably linked to down-regulated transcription of TLR2 gene, and with diminished TLR2 level. Under such conditions, the pro-inflammatory cytokines are partially repressed. Thus, the inflammation and hepatocytes damage were relieved by mutation involving the TLR2 promotor domain [16]. Accordingly, to observe the effect of TLR2 polymorphism on cytokine production in CHB patients, we analyzed the correlation between the obtained genotypes of rs3804099 SNP of TLR2 and serum IL-6 values. A significant relation was noted amongst serum IL-6 levels and TLR genotypes; where inactive carriers with the mutant TT genotype had lower IL-6 levels and for allelic distribution it was clear that T allele correlated with lower cytokine level.

Piñero et al. noticed that IL-6, TNF-α, and serum levels of nitric oxide were considerably lower in all patients with variant TLR genotyps. Cytokine levels were significantly less upregulated in response to specific TLR-ligands in patients with all variant vs. wild type TLR genotypes. They reported that SNPs in TLR-2, TLR-4 and TLR-9 were associated with a significant decrease in MyD88 and NFκB (nuclear factor-kB) compared to those with wild type alleles after stimulation with their respective specific ligands [39].

Over and above in the current study, the multivariate regression analysis demonstrates that the rs3804099 TLR2 mutation was inversely associated with hepatitis activity as found between inactive carriers and active CHB patients as well as between active CHB patients and those with HBV-related liver cirrhosis. Similarly, Lin et al. has reported in their case–control study in a Han Chinese population that the logistic regression results showed an inverse association between the mutations in rs3804099 of TLR-2 and hepatitis B activity as evaluated by METAVIR [16]. However, Isogawa et al. demonstrated that, in mice in vivo, TLR2 activation was not able to restrain HBV infection [40]. These finding displayed that the concept of TLR2 activation as a protective factor is yet challenging. There were some limitations for our study. First, the number of participants was limited. Second, the participants were only selected from the Menoufia university hospitals population. Third, our cases were chosen from a hospital, and the controls were from communities, which may not be representative of the general population.

## Conclusions

Our findings propose on one side, a positive association between TLR2 polymorphisms and hepatitis B activity, and, on the other, the valuable role of IL-6 a good marker of disease progression in chronic HBV-infected patients. With the guide achieved from this study, it is potential to predict patients who are more like enough to progress to an advanced stage of CHB so as to provide targeted interventions. The underlying mechanism between cytokines and TLR2 polymorphisms has likewise to be specified. Large population-based prospective studies are warranted to further appraise the impact of TLR2 SNPs as well as different cytokines on HBV disease activity and outcomes.

## Data Availability

All data are presented in the main manuscript.

## Abbreviations

AFP: α-Fetoprotein
ALT: Aspartate transaminase
AST: Alanine transaminase
CHB: Chronic hepatitis B
DBIL: Direct bilirubin
DNA: Deoxyribonucleic acid
ELISA: Enzyme-linked immunosorbent assay
HB: Hemoglobin concentration
HbsAg: Hepatitis B surface antigen
HBV: Hepatitis B virus
HCV: Hepatitis C virus
HIV: Human immunodeficiency virus
INR: International normalized ratio
SNPs: Single nucleotide polymorphisms
TBIL: Total bilirubin
TLR: Toll-like receptors
WBCs: White blood cells
